# Evaluation of 3-Dimensional Superimposition Techniques on Various Skeletal Structures of the Head Using Surface Models

**DOI:** 10.1371/journal.pone.0118810

**Published:** 2015-02-23

**Authors:** Nikolaos Gkantidis, Michael Schauseil, Pawel Pazera, Berna Zorkun, Christos Katsaros, Björn Ludwig

**Affiliations:** 1 Department of Orthodontics and Dentofacial Orthopedics, University of Bern, Bern, Switzerland; 2 Department of Orthodontics, University of Marburg, Marburg, Germany; 3 Department of Orthodontics, Cumhuriyet University, Sivas, Turkey; 4 Private orthodontic office, Traben-Trarbach, Germany; 5 Department of Orthodontics, University of Saarland, Homburg/Saar, Germany; Max Planck Institute for Evolutionary Anthropology, GERMANY

## Abstract

**Objectives:**

To test the applicability, accuracy, precision, and reproducibility of various 3D superimposition techniques for radiographic data, transformed to triangulated surface data.

**Methods:**

Five superimposition techniques (3P: three-point registration; AC: anterior cranial base; AC + F: anterior cranial base + foramen magnum; BZ: both zygomatic arches; 1Z: one zygomatic arch) were tested using eight pairs of pre-existing CT data (pre- and post-treatment). These were obtained from non-growing orthodontic patients treated with rapid maxillary expansion. All datasets were superimposed by three operators independently, who repeated the whole procedure one month later. Accuracy was assessed by the distance (D) between superimposed datasets on three form-stable anatomical areas, located on the anterior cranial base and the foramen magnum. Precision and reproducibility were assessed using the distances between models at four specific landmarks. Non parametric multivariate models and Bland-Altman difference plots were used for analyses.

**Results:**

There was no difference among operators or between time points on the accuracy of each superimposition technique (p>0.05). The AC + F technique was the most accurate (D<0.17 mm), as expected, followed by AC and BZ superimpositions that presented similar level of accuracy (D<0.5 mm). 3P and 1Z were the least accurate superimpositions (0.79<D<1.76 mm, p<0.005). Although there was no difference among operators or between time points on the precision of each superimposition technique (p>0.05), the detected structural changes differed significantly between different techniques (p<0.05). Bland-Altman difference plots showed that BZ superimposition was comparable to AC, though it presented slightly higher random error.

**Conclusions:**

Superimposition of 3D datasets using surface models created from voxel data can provide accurate, precise, and reproducible results, offering also high efficiency and increased post-processing capabilities. In the present study population, the BZ superimposition was comparable to AC, with the added advantage of being applicable to scans with a smaller field of view.

## Introduction

Researchers in the fields of craniofacial development and orthopedics have always been interested in quantifying the effect of treatment on craniofacial morphology. Historically, superimpositions of cephalometric radiographs have been used to differentiate between growth and treatment effects. However, bone modeling and remodeling make the superimposition of radiographs challenging since they constantly alter the morphology of craniofacial skeletal structures. In the past, Tantalum implants were used in an attempt to identify form-stable anatomical structures (size and shape unaffected by treatment and/or growth) that could be used as references [1,2]. The assessment of size and shape changes using conventional 2D (dimensions) radiographs also raises the important issue of reducing a 3D object to a 2D image. The inherent information in this simplified image can be further confounded due to the reference structures used to consistently superimpose and compare serial radiographs [3–5].

In recent years, 3D imaging techniques have been widely used in maxillofacial surgery [6–8], dental and orthodontic implantology [9], as well as in various other medical disciplines [10]. The 3D model of a patient can be superimposed on other such models from the same or other patients. This can help identify treatment goals, choose treatment modalities, predict treatment result, and evaluate treatment and/or growth changes.

Various techniques have been reported for superimposition of 3D datasets derived from either conventional computed tomography (CT) or lower radiation cone beam CT (CBCT) images [8,11–17]. These include landmark-based superimposition, surface-based superimposition, or voxel-based superimposition of form-stable anatomical structures [11–14]. Among others, the validity of the first two superimposition techniques depends on the accuracy of landmark identification and on the precision of the 3D surface models, respectively [11,18,19]. The voxel-based superimposition was developed in an attempt to overcome these limitations [15,16]. This requires file formats that contain 3D volume information as lattices of voxel data, each voxel describing the radiodensity of the tissues that it represents [11]. Relevant software registers serial 3D datasets according to the grey level intensity of each voxel in corresponding reference structures. In most available software, the 3D differences of the superimposed models are usually translated into 2D color codes that represent the distance between corresponding points. However, the quantification of the structural change is not always easy. This process was until recently very sensitive and time consuming and required very powerful hardware systems [14–17]. A recent study reported an efficient technique for voxel-based superimposition of bone structures captured by CBCT scans, which still required 30–40 min to be accomplished [16]. Also, the form of data (e.g. CT or CBCT and voxel size), the segmentation process, the transformation model and various other factors can violate the assumption of correspondence in voxel intensities [5].

Surface-based registration could qualify as a valid alternative of the common used voxel-based approaches in medical imaging. STL (Standard Tessellation Language) is an open source surface-based format, like DICOM (Digital Imaging and Communications in Medicine) for voxel data, and is easily accessible through most commercial and freeware software applications. Such surface models have been widely used in industry, particularly in engineering and architecture, for rapid prototyping and computer-aided manufacturing. These 3D datasets allow for easy information exchange and communication among scientists. For example, integration into commonly used applications, such as Microsoft Office (Microsoft, Redmond, Washington, USA), and export into other widely used freeware formats, such as a 3D PDF document (Adobe Systems Inc. San Jose, California, USA) [20], are easily performed. Surface models do not contain any volume data, but instead use 3D surface data that are different from the data obtained from a CT or CBCT machine. Namely, this is a triangular representation of a 3D surface geometry. Also in this case, the form of data, the surface preprocessing (e.g. smoothing, segmentation), the transformation model and the choice of reference structures should be considered as potential sources of error when superimposing surface models.

In this study we utilized existing software applications for the superimposition of serial 3D surface models of craniofacial hard tissue structures, obtained from CT images. We aimed to identify a simple, efficient, and accurate way to detect, visualize, and quantify skeletal differences and investigate its applicability in smaller field of view scans. For this reason, we assessed five different superimposition techniques, using various anatomical structures as references. One of them, the superimposition on the anterior cranial base + foramen magnum, was considered the gold standard technique, because of the anatomical form stability [21,22] and the location of the reference structures. This technique requires large field of view images, which implies increased radiation dose and cost [23,24]. Thus, it was used for comparisons with other techniques, in an attempt to identify alternatives that give similar results, but are applicable to smaller field of view scans.

## Material and Methods

The material for this study consisted of existing pre- and post-treatment CT scans (Philips Brilliance 16 CT Scanner, tube voltage: 120 kV, tube current: 293mA, field of view/FOV: 21 cm transversal x 21 cm anteroposterior x 12 cm height, 2.5 sec exposure time, 0.8 mm slice thickness, 0.4 mm spacing between slices, 0.8 mm^3^ voxel size, 16 lp/cm spatial resolution) of eight young adult patients (median: 16.2, range: 15.1, 22.9 years; 2M, 6F). They had severe maxillary transverse deficiency, treated with rapid maxillary expansion performed by a mini-implant supported device [25]. The first scan was obtained just before placement of the appliance and the second one at the end of the activation period at a median of 15 days later (range: 10, 23). The appliance was activated to expand the maxilla by approximately 0.6 mm per day. Witnessed oral informed consent was obtained from all participants and their parents/guardians, in case of non-adult patients, regarding data acquisition, storage, and potential use for research. Ethical approval for the use of retrospective data was obtained by the Medical Ethical Commission of Saar, Germany (170/12). According to the Commission, witnessed oral informed consent was adequate and there was no relevant ethical or legal issue.

For analysis and superimposition of 3D images we used an Apple Macbook Pro 8.1 (Apple, Cupertino, California, USA) with a 2.8 GHz Intel Core i7 processor (Santa Clara, California, USA) and 8 GB, 1333 Mhz, DDR3 RAM. The operating system was MacOS X 10.7.2 and Windows 7 by implementation of emulation software. The primary software used in this study was Geomagic Qualify 2012 for Windows (Geomagic GmbH, Stuttgart, Germany) [26] that was adapted for orthodontic use.

### Reference structures

**Fig 1:**
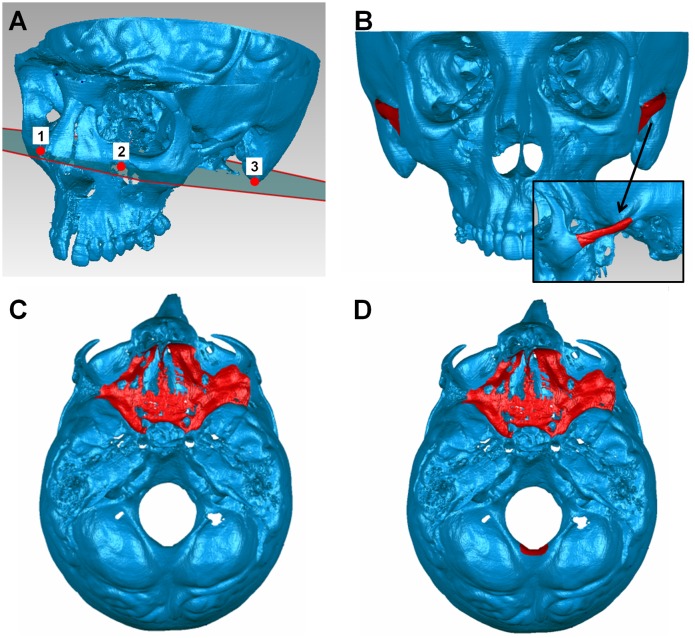
Reference points/structures used for each superimposition technique. (A) Points used for the three-point registration technique (numbered red dots): the most superior point of the infraorbital foramen on the left and right side and the lowest point of the mastoid process on the left side. (B) The used part of the zygomatic arch (red color) extended from the most anterior point of the zygomaticomaxillary suture to the junction of the arch with the main part of the temporal bone. (C) The used part of the anterior cranial base (red color) included the body and small wing of the sphenoid bone and part of the bottom of the anterior cranial fossa (the sella turcica, the orbital upper wall, the sinuses upper walls, and the cribriform plate and crista galli of the ethmoid bone were not included). (D) The used part of the foramen magnum included the middle posterior part of the edge of the foramen magnum with the posterior cranial fossa, having approximately a height of 1 cm and a width of 1.5 cm (red color).

The available 3D datasets from each patient were superimposed using five different reference points/structures that have been widely used both for clinical and research purposes. These included the following:

*a) Three-point registration (3P)*: Most commercially available software programs offer the possibility of manual or automatic superimposition of 3D datasets using three anatomical landmarks [11] (Fig. 1A).

*b) One zygomatic arch (1Z)*: This superimposition has been recommended for scans with small FOV, where the base of skull or both zygomatic arches are not available [16]. We used the left zygomatic arch in all cases (Fig. 1B).

*c) Both zygomatic arches (BZ)*: This is an alternative technique for scans with small FOV (Fig. 1B) [16].

*d) Anterior cranial base (AC)*: This has been considered for several years a standard reference for cephalometric superimposition of serial radiographs, even in growing patients (Fig. 1C) [21].

*e) Anterior cranial base + foramen magnum (AC + F)*: The foramen magnum region was recommended in the past as a valid superimposition reference even in early growth stages [22], although it did not gain much attention afterwards. In the present study, this region, together with the anterior cranial base (Fig. 1D), was considered the gold standard superimposition reference used for comparisons with alternatives. This technique was expected to offer the highest level of accuracy in our sample, because of the location and the stable anatomical form of the reference structures [21,22] suggested by developmental and treatment related parameters.

### Superimposition workflow

Serial datasets from each patient were superimposed through a semi-automatic surface matching technique, using each of the above reference structures [27]. The reference structures were selected manually and did not have to be identical in the two superimposed models, neither in content nor in extent [28,29]. However, for consistency reasons, the operators were instructed to select similar reference areas between different models and patients, as described in Fig. 1. The workflow of the superimposition of two serial DICOM datasets (T0 = pre-treatment and T1 = post-treatment) is described below in six steps, which required approximately 25 minutes.

***Step 1*** *—Conversion of DICOM to STL*: This conversion can be undertaken in most operating systems that run DICOM viewers [30,31]. We tested the internal DICOM converter of the Geomagic Qualify 2012 software to avoid multitasking, but this required quite a powerful hardware system to work properly. Thus, we used Osirix [30], a well-tested freeware option [32–35]. Tai et al. [17] demonstrated that conversion of datasets from voxel into polygonal surface data is dimensionally stable, independent of the software used.

The two DICOM datasets (T0 and T1) were opened in Osirix [30] and after setting the desired threshold and quality for bone structures in the surface rendering mode (maximum analysis, noise reduction not selected, lower threshold value: between 300 to 500 Hounsfield Units/HU per pixel), 3D triangular meshes were created. For this, the software creates the iso-surfaces and afterwards it employs a marching cubes algorithm [36] to create 3D-polygons. The range of included HU values was selected automatically by the software after selecting the ‘‘bone” option (usually 500 HU), and adjusted manually afterwards in case of unsatisfactory visualization of the maxillary bone. In all cases, the same threshold value was used for each pair of patient data (T0 and T1), but not for different patients. The STL-models were created once by each operator and were then used for all five superimposition techniques.

***Step 2*** *—Initial model processing*: The 3D surface models (T0 and T1) were imported into Geomagic Qualify 2012 and were roughly trimmed to remove unnecessary information. Small artifacts identified as independent polygons, such as those caused by metallic appliances, were automatically removed using the ‘manifold’ function.

**Fig 2:**
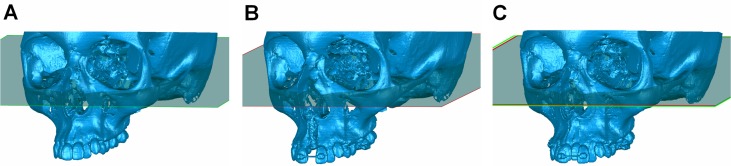
Initial manual superimposition of two serial datasets prior to final superimposition. Initial manual superimposition of the two models on certain stable structures or points was performed to shorten the time needed for the subsequent automatic superimposition. Thus, pairs of models were oriented and roughly registered by using the FH-plane and a line delimited by the infraorbital foramina as shown in the images. (A) Pre-treatment model, (B) post-treatment model, and (C) initially registered models.

***Step 3*** *—First registration*: Initial manual superimposition of the two models was performed to shorten the time needed for the subsequent automatic superimposition. Thus, pairs of models were oriented and roughly registered by using the FH-plane and a line delimited by the infraorbital foramina (Fig. 2).

***Step 4*** *—Final registration*: Final registration was performed using the ‘‘Best fit alignment” option in Geomagic Qualify 2012. After defining the reference dataset (T0), setting the precision of the registration to at least 0.3 mm (tolerance type: ‘‘3D Deviation”) and the number of polygons used for surface representation to the maximum of 100.000, corresponding polygons of the selected reference areas were automatically superimposed. Superimposition was performed using an iterative closest point algorithm, which is called robust iterative closest point (RICP) [29]. The distances between the T0 and the T1 models were minimized using the point-to-plane method [28,37].

Congruence between specific corresponding structures was calculated at this stage, as described below, for testing the accuracy of the procedure.

***Step 5*** *—Trimming*: Following superimposition, the STL models were further ‘trimmed’ in all three dimensions, to remove unnecessary information and facilitate subsequent analysis.

**Fig 3:**
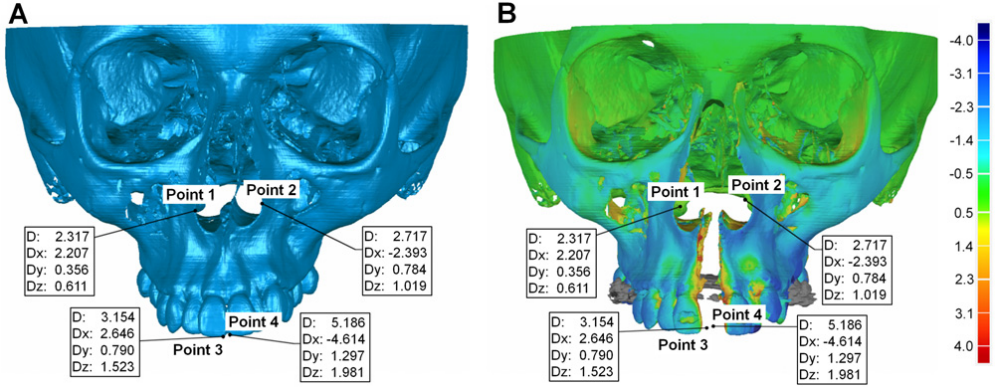
Visualization of a superimposition on the anterior cranial base + foramen magnum. Frontal view of the superimposition of two serial 3D datasets (T0 and T1) on the anterior cranial base + foramen magnum. Quantification of structural changes at four specific points, overall (D) and in all three planes of space (Dx, Dy, Dz), in (A) the pre- and (B) the post-treatment model, where color-coding was also used. These were the most lateral points of the piriform aperture on the left and right side (points 1 and 2) and the most labial point of the mesio-incisal corner of each central incisor (points 3 and 4). All values are in mm. The grey area in the color-coded model is due to the metal artefact produced by the Hyrax appliance. The software was unable to compare corresponding polygons at that site, because the appliance was present only in one model (T1).

***Step 6*** *—Superimposition and 3D analysis*: The distances between corresponding areas of T0 and T1 were color-coded on the superimposed models for visualization of the result. The distance between specific points of interest was quantified overall and in all three planes of space (Fig. 3).

### Accuracy, precision, and reproducibility of Superimposition

**Fig 4:**
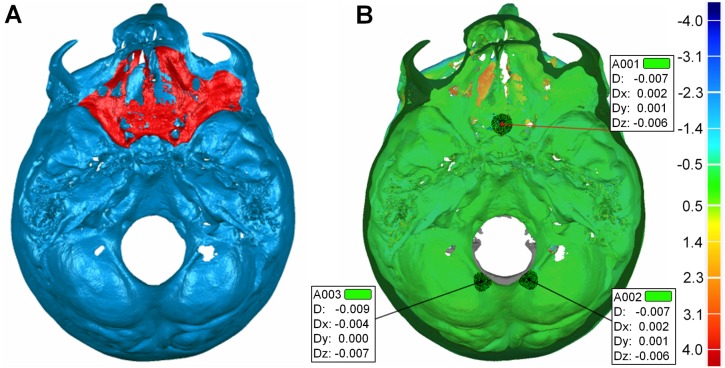
Test of the accuracy of each superimposition technique. After superimposition of two models (T0 and T1) in a certain area, such as (A) the anterior cranial base, accuracy was verified by measuring the distance between the two models overall (D) and in all three planes of space (Dx, Dy, Dz) on (B) three 5 mm^2^ circular areas: 1. anterior surface of the sella turcica and Walker’s point, 2. posterior right side of the foramen magnum, and 3. posterior left side of the foramen magnum. These structures are considered form-stable in our sample and thus complete congruence of the two models (T0 and T1) is expected at the specific sites in case of successful superimposition.

Three operators performed all five superimpositions for each of the eight patients, independently, after being calibrated on the procedure. Following a 3D superimposition, the software allows for the assessment of its accuracy by measuring the congruence of specific corresponding areas (step 4 of the superimposition workflow). Accuracy was verified on three 5 mm^2^ circular areas: 1. anterior surface of the sella turcica and Walker’s point, 2. posterior right side of the foramen magnum, and 3. posterior left side of the foramen magnum (Fig. 4). In a preliminary study, the three operators performed the measurement of accuracy in 5 mm^2^ and 15 mm^2^ areas and no significant differences were detected. After selecting one such area at the post-treatment model (T1, the coloured result model) the software automatically compares its congruence to the corresponding area (the area in shortest distance) of the other model (T0). These structures are considered to have stable anatomical form in our study group, both for developmental and treatment-related reasons. Thus, complete congruence of the two models is expected at the specific sites. These sites are neighboring to the anterior cranial base and the foramen magnum and thus increased accuracy is expected for our gold standard technique (AC + F) also for this reason.

As a measure of accuracy we used the overall deviation (D; the mean Euclidean distance between corresponding polygons) of two superimposed datasets at three specific areas, divided by three. Low accuracy values imply high congruence of these areas in all three planes, and thus increased accuracy of the superimposition.

To test the precision of each superimposition technique in measuring structural changes between T0 and T1, the distances of the models at four corresponding points/landmarks were analyzed. These were the most lateral points of the piriform aperture, on the left and right side, and the most labial point of the mesio-incisal corner of each central incisor (Fig. 3). These parameters depend on the accuracy of each superimposition technique and represent the main outcome of clinical importance.

All three operators performed the whole procedure and repeated all measurements one month later to test the reproducibility of each technique.

As an additional control, we tested the effect of only the registration procedure on accuracy and precision of each technique by superimposing duplicated pre-treatment datasets of each patient. This way we removed the potential effect of medical treatment on results. For this reason, one operator superimposed each pair of datasets after modifying the location information on the duplicated models.

### Statistical analysis

The statistical analysis was carried out by using the SPSS (v.20.0, SPSS Inc., U.S.A), PERMANOVA [38,39], and PERMDISP [40] software.

Data were tested for normality through the Shapiro-Wilk test and were not normally distributed in all cases (SPSS). Thus, non-parametric statistics were applied. Differences in the measured variables were evaluated using permutational multivariate analysis of variance (MANOVA) with factorial mixed effects models. Pair-wise *a posteriori* comparisons of levels for single factors, including within individual levels of other factors in the case of significant interactions, were performed when significant differences were detected by the multivariate model. For accuracy testing three crossed factors and their possible interactions were analyzed: superimposition technique (fixed factor; 5 techniques), operator (random factor; 3 operators), and time (fixed factor; 2 time points). For precision testing four crossed factors and their possible interactions were analyzed: superimposition technique (fixed factor; 5 techniques), point (fixed factor; 4 points), operator (random factor; 3 operators), and time (fixed factor; 2 time points).

Permutational MANOVA was done on Euclidean distances calculated from log10 transformed data. The P-value was calculated through unrestricted permutations of raw data with 9999 random permutations. In cases when there were few unique permutations possible Monte Carlo asymptotic p-value was used instead [38] (PERMANOVA). Results were similar when the analysis was performed on Bray-Curtis distances calculated from fourth-root transformed data under the full model (S1 Table) or when using permutation of residuals under a reduced model (S2 Table).

Permutational analysis of multivariate dispersions (PERMDISP) were used to determine whether potential differences between any pair of groups were due to location, dispersion or a combination of the above.

In all cases, a two-sided significance test was carried out at an alpha level of 0.05. The level of significance used for the study was set at 0.05. Bonferroni correction was applied for pair-wise *a posteriori* multiple comparison tests.

To overcome potential limitations of methods described above [41], the Bland-Altman method (difference plot) [42] was also used to evaluate agreement between the gold standard technique and all other techniques.

## Results

The main effects of different operators and time points on accuracy measurements were not significant, implying sufficient intra- and inter-operator agreement. No significant interaction was detected apart from a minor interaction (small effect size) of superimposition and time that was not further explored.

Different superimposition techniques showed significant differences in accuracy. Pair-wise *a posteriori* tests between superimposition techniques showed that all techniques differed from each other (p < 0.005), except for the AC and BZ superimpositions (Table 1). Permutational analysis of multivariate dispersions showed that differences were not due to dispersion (d.f. = 4, F = 4.81, p = 0.0843, R^2^ = 0.06) and thus they were due to location. The AC + F was the most accurate technique (as expected for reasons described above), followed by AC and BZ superimpositions that presented similar level of accuracy, but approximately four times reduced relative to AC + F. 3P and 1Z superimpositions were the least accurate, in decreasing order (Table 2).

**Table 1:** Non parametric MANOVA on accuracy measurements (deviation between structures).

Source	d.f.	SS	MS	F	p
Superimposition	4	33.07	8.27	169.67	0.0002*
Operator	2	0.15	0.07	0.71	0.4899
Time	1	0.56	0.57	48.67	0.0658
Superimposition x Operator	8	0.39	0.05	0.47	0.8711
Superimposition x Time	4	1.00	0.25	5.20	0.0232*
Operator x Time	2	0.02	0.01	0.11	0.8909
Superimposition x Operator x Time	8	0.39	0.05	0.47	0.8787
Residual	210	21.58	0.10		
Total	239	57.16			

Comparison^1^	t	p
3P vs. AC	10.16	0.0008*
3P vs. AC + F	25.10	0.0001*
3P vs. BZ	5.57	0.0050*
3P vs. 1Z	6.64	0.0036*
AC vs. AC + F	15.69	0.0001*
AC vs. BZ	0.87	0.4345
AC vs. 1Z	14.87	0.0003*
AC + F vs. BZ	8.95	0.0010*
AC + F vs. 1Z	28.56	0.0001*
BZ vs. 1Z	7.72	0.0010*

Three crossed factors and their possible interactions were analyzed: superimposition technique (fixed factor; 5 techniques), operator (random factor; 3 operators), and time (fixed factor; 2 time points). Data were transformed to log10(x). Analysis was based on Euclidean distances. Unrestricted permutation of raw data using correct permutable units was performed. No. of permutations used = 9999. R^2^ = 0.62%.

^1^Pair-wise *a posteriori* tests among superimposition techniques using the *t*-statistic (p < 0.005; Bonferroni correction applied; Monte Carlo asymptotic p-value). Operators and time points were ignored in the pair-wise tests.

* denotes statistical significance.

3P: three-point registration; AC: anterior cranial base; AC + F: anterior cranial base + foramen magnum; BZ: both zygomatic arches; 1Z: one zygomatic arch

**Table 2:** Accuracy values of each superimposition technique.

	3P	AC	AC + F	BZ	1Z
**Operator 1**	1.01 (0.68, 1.31)	0.42 (0.36, 0.65)	0.11 (0.09, 0.17)	0.31 (0.23, 0.61)	1.44 (1.02, 2.17)
**Operator 2**	1.01 (0.71, 1.66)	0.35 (0.21, 1.16)	0.07 (0.04, 0.09)	0.44 (0.27, 0.96)	1.42 (0.91, 2.21)
**Operator 3**	0.79 (0.50, 1.03)	0.52 (0.38, 0.83)	0.09 (0.04, 0.14)	0.57 (0.32, 1.50)	1.76 (1.04, 2.32)

Values represent median (interquartile range) of overall deviation (D) between corresponding form-stable structures in millimeters (n = 8 patients x 1 time point x 1 value = 8).

3P: three-point registration; AC: anterior cranial base; AC + F: anterior cranial base + foramen magnum; BZ: both zygomatic arches; 1Z: one zygomatic arch

The precision of all superimposition techniques was high since there were no significant differences among operators on measured structural changes. The reproducibility of all techniques was also sufficient since there were no significant differences between repeated measurements. However, the detected structural changes differed significantly when comparing the superimposition techniques to each other. The existing differences were also modified according to the tested point (Table 3). Permutational analysis of multivariate dispersions showed that a small amount of these differences was due to dispersion (d.f. = 4, F = 9.6049, p = 0.0001, R^2^ = 0.14) and thus differences were mainly due to location.

**Table 3:** Non parametric MANOVA on precision measurements (distances between points).

Source	df	SS	MS	F	P
Superimposition	4	8.40	2.10	284.35	0.0001*
Point	3	3.39	1.13	153.90	0.0001*
Operator	2	0.03	0.01	0.41	0.6610
Time	1	0.00	0.00	0.00	0.8645
Superimposition x Point	12	12.73	1.06	135.46	0.0001*
Superimposition x Operator	8	0.06	0.01	0.21	0.9889
Superimposition x Time	4	0.07	0.02	1.97	0.1937
Point x Operator	6	0.04	0.01	0.21	0.9764
Point x Time	3	0.04	0.01	0.54	0.6788
Operator x Time	2	0.12	0.06	1.75	0.1772
Superimposition x Point x Operator	24	0.19	0.01	0.22	1.0000
Superimposition x Point x Time	12	0.07	0.01	0.29	0.9875
Superimposition x Operator x Time	8	0.07	0.01	0.25	0.9813
Point x Operator x Time	6	0.16	0.03	0.73	0.6286
Superimposition x Point x Operator x Time	24	0.45	0.02	0.53	0.9726
Residual	840	30.01	0.04		
Total	959	55.84			

Comparison^1^	t—Point 1	t—Point 2	t—Point 3	t—Point 4
3P vs. AC	20.05*	2.40	2.140	1.96
3P vs. AC + F	4.11	17.64*	8.47*	19.75*
3P vs. BZ	13.71*	6.26*	28.02*	8.18*
3P vs. 1Z	17.36*	13.75*	11.99*	0.92
AC vs. AC + F	20.82*	13.74*	8.14*	17.06*
AC vs. BZ	10.41*	8.60*	48.54*	6.97*
AC vs. 1Z	1.10	16.31*	13.75*	1.82
AC + F vs. BZ	15.95*	27.970*	9.14*	0.89
AC + F vs. 1Z	19.03*	68.48*	0.10	54.68*
BZ vs. 1Z	7.06*	6.29*	16.87*	8.60*

Four crossed factors and their possible interactions were analyzed: superimposition technique (fixed factor; 5 techniques), point (fixed factor; 4 points), operator (random factor; 3 operators), and time (fixed factor; 2 time points). Data were transformed to log10(x). Analysis was based on Euclidean distances. Unrestricted permutation of raw data using correct permutable units was performed. No. of permutations used = 9999. R^2^ = 0.46%.

^1^Pair-wise *a posteriori* tests among superimposition techniques within each point using the *t*-statistic (p < 0.005; Bonferroni correction applied; Monte Carlo asymptotic p-value). Operators and time points were ignored in the pair-wise tests.

* denotes statistical significance.

3P: three-point registration; AC: anterior cranial base; AC + F: anterior cranial base + foramen magnum; BZ: both zygomatic arches; 1Z: one zygomatic arch

**Fig 5:**
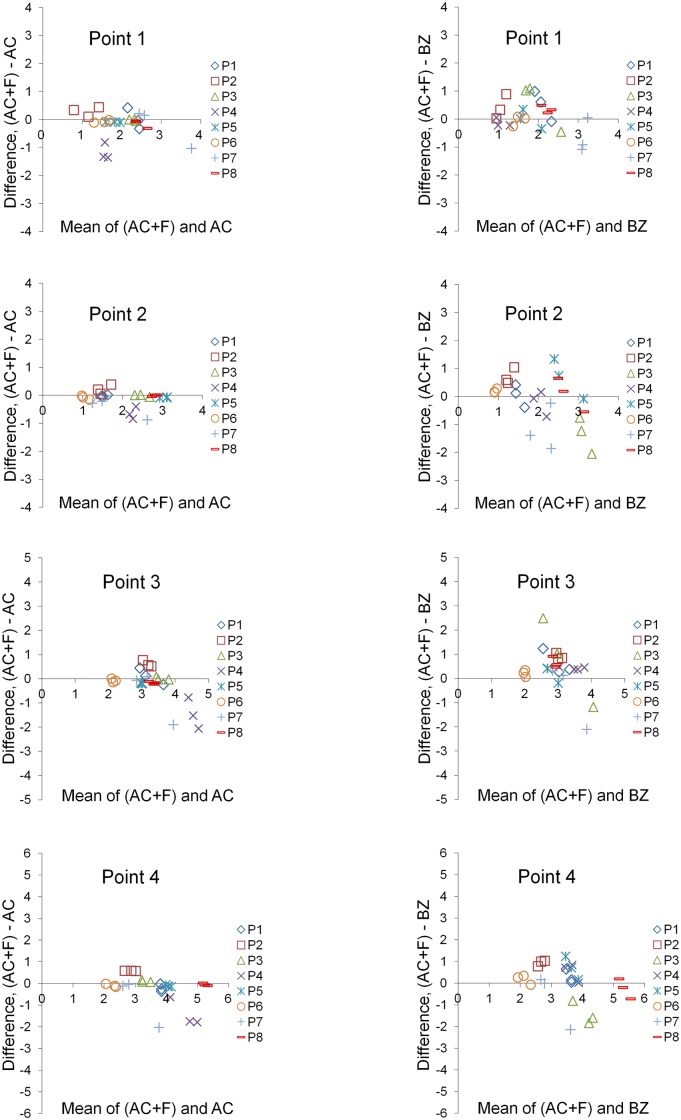
Differences of the AC and BZ superimposition techniques from the gold standard technique. Bland-Altman plots of differences of the anterior cranial base (AC) or both zygomatic arches (BZ) superimposition techniques from the gold standard superimposition technique (anterior cranial base + foramen magnum: AC+F). These consider the measured structural changes induced by treatment at four specific points, measured by each operator, for all eight patients (P). The axes length represents the true range of observed values of structural changes.

**Fig 6:**
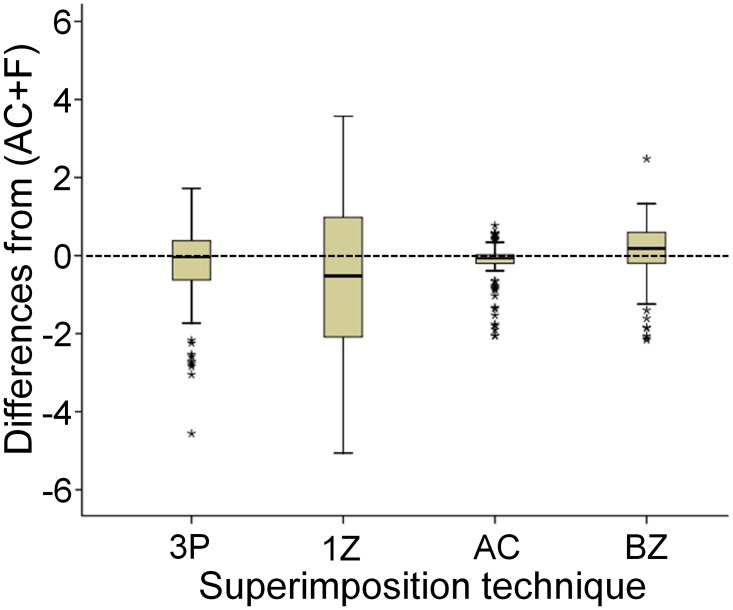
Differences in measured structural changes by each technique from the gold standard. Boxplots showing the medians and quartile distances (boxes: 25^th^ and 75^th^ percentage) of the differences in measured structural changes by each superimposition technique from the gold standard. The t-bars represent the smallest and largest value, provided there are no outliers. Outliers are considered values which lie above 1 ½ box lengths outside of the box and are shown as asterisks. (3P: three-point registration; AC: anterior cranial base; AC + F: anterior cranial base + foramen magnum; BZ: both zygomatic arches; 1Z: one zygomatic arch).

Bland-Altman plots of differences of the AC from the gold standard (AC + F) superimposition showed that the measured structural changes were quite similar in most cases (median: -0.07; IQR: -0.20, 0.02; 95% CI: -0.31, -0.08). The higher differences were mostly in negative direction and mainly concerned two specific patients, implying a systematic error restricted to these cases (Figs. 5 & 6). Regarding the BZ superimposition, the measured structural changes were also quite similar to AC + F in most cases (median: 0.18; IQR: -0.20, 0.60; 95% CI: -0.06, 0.27). The distribution of differences tended to vary slightly more compared to the AC superimposition. The higher differences did not have a certain direction and were not attributed to selective patients (Figs. 5 & 6). There was no evidence that the extent of difference between techniques was related to the extent of structural changes in either case (Fig. 5). Thus, both superimposition techniques can be considered acceptable, though Bland-Altman plots reveal higher random error of the BZ superimposition. On the contrary, small systematic error may be evident in the AC superimposition (Fig. 6).

**Fig 7:**
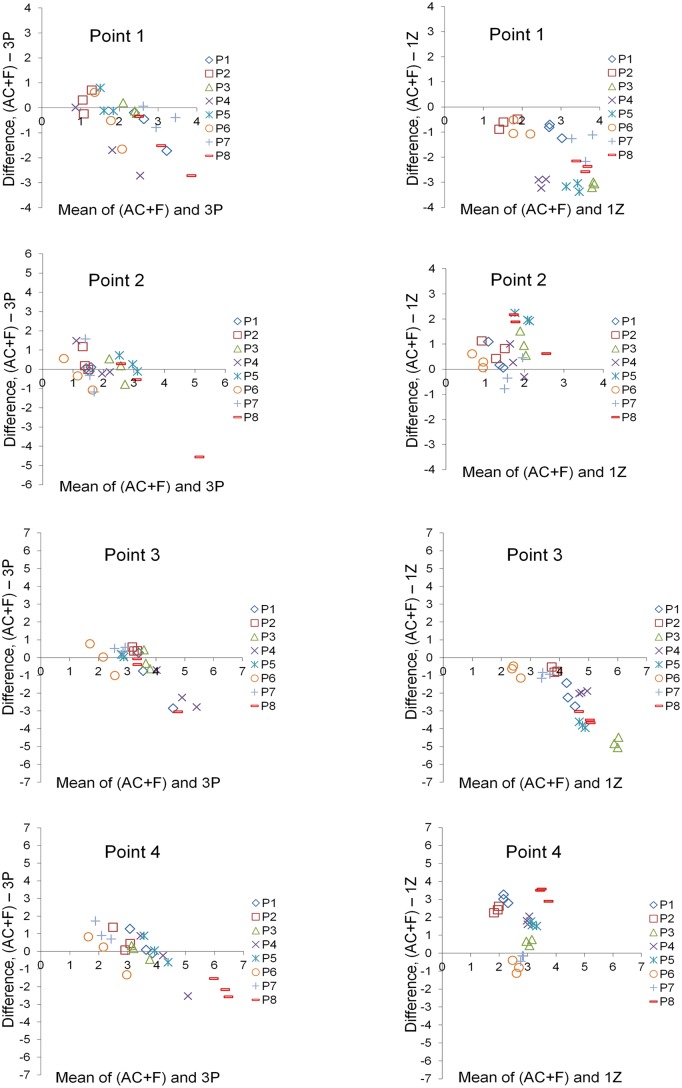
Differences of the 3P and 1Z superimposition techniques from the gold standard technique. Bland-Altman plots of differences of the three-point (3P) or one zygomatic arch (1Z) superimposition techniques from the gold standard superimposition technique (anterior cranial base + foramen magnum: AC+F). These consider the measured structural changes induced by treatment at four specific points of interest, measured by each operator, for all eight patients (P). The axes length represents the true range of observed values of structural changes.

Bland-Altman plots regarding 1Z superimposition showed that in many cases the measured structural changes were quite different from AC + F (median: -0.52; IQR: -2.12, 0.99; 95% CI: -0.92, -0.08). Furthermore, there was a clear systematic error depending on the point tested (Figs. 6 & 7). 3P superimposition resulted in high differences from AC + F in part of the tested cases (median: -0.04; IQR: -0.63, 0.38; 95% CI: -0.51, -0.05). In these cases, a tendency for systematic error in the direction of structural changes was evident (Fig. 6). Furthermore, both techniques showed a tendency for increased differences with increasing extent of structural changes (Fig. 7). Therefore, both 1Z and 3P techniques can be considered less suitable due to decreased accuracy and precision.

When duplicated pre-treatment models were superimposed, the accuracy of all techniques was excellent (D < 0.001 mm) apart from the 3P technique (D; median: 0.53, range: 0.04, 1.55). Precision also followed a similar pattern. The distances between corresponding points were less than 0.001 mm with all techniques apart from the 3P superimposition (median: 0.76, range: 0.28, 2.20). Thus, the duplicated models were perfectly registered with all techniques, whereas 3P showed considerable inaccuracy and imprecision, probably due to inconsistency in landmark identification.

## Discussion

In this study we presented an efficient, reproducible, precise, and accurate superimposition technique for radiographic data of craniofacial skeletal structures. We demonstrated that 3D surface models, which offer considerable post-processing options, can be used as an alternative to the common used voxel-based models. We tested various superimposition references, considering the AC + F as the gold standard reference. Indeed, it provided the most accurate and precise superimpositions due to the anatomical form stability of these structures that were not affected by treatment or growth and the measurement of accuracy in/near these regions. The important finding of the study was that in our sample AC and BZ superimpositions presented similar levels of accuracy and precision, which can be considered acceptable both for clinical and research purposes. BZ superimposition has the additional advantage of being applicable in smaller FOV scans, which require less radiation dose and cost.

The anterior cranial base attains final size and shape early during development (around 8 years of age), with minimal changes occurring thereafter [43]. Therefore, it remains a standard reference for optimal superimpositions even in growing patients [14,21], although it requires larger FOV scans. Our results suggest that BZ can be a viable alternative to AC superimposition, even when the zygomatic arches are largely affected by treatment. This technique is applicable to scans with reduced FOV that can be obtained with approximately half of the radiation needed for the large FOV examinations. Exposure to radiation is a major concern when ionizing radiation is used for diagnostic purposes and a careful risk/benefit analysis should always be considered before image acquisition [23,24]. Our study sample had limited growth potential (late adolescence to early adulthood) and the observation period was small, but the applied treatment caused extensive changes of a wide range of craniofacial structures, at least in position and orientation. So, reduction of FOV by using BZ superimposition might also be feasible in growing patients, although this remains to be tested.

The clinician or researcher should be aware of the limitations of each technique in order to choose the one that best fits the data and the question under study. The important issue is if and how different registration techniques influence the conclusions drawn in the evaluation of structural changes. Bland-Altman difference plots for measured structural changes revealed slightly increased random error of BZ superimposition. This could be due to the significant changes in the zygomatic arches imposed by treatment. However, even the AC superimposition, which has traditionally been considered a standard for cephalometric superimpositions [14,16,44–46], presented a degree of random and systematic error in few cases. The random error can be attributed to small inaccuracies of the 3D model construction [47] or to variations in landmark identification [18,19]. A potential source of the systematic error could be the inadequate posterior and lateral control in AC superimpositions. Both types of error of AC or BZ superimpositions are not large enough to question their application in clinical practice. In the vast majority of cases differences in the measured structural changes are below 1 mm. Such small differences are not considered important by doctors when evaluating craniomaxillofacial surgery result [48,49] and are not perceived as important by patients when evaluating facial esthetics [50,51]. However, it is important to note that neither of these limitations was evident by correlation analyses (S3 Table) or comparisons of means (S4 Table), which are regularly used in the relevant literature for testing such hypotheses [16]. This underlines the need for appropriate statistical methods, such as the Bland-Altman method, in studies comparing methods of measurement in order to avoid misleading results [41].

On the other hand, 3P and 1Z superimpositions can be considered inappropriate, since they are less accurate and sometimes present quite high errors. This is in contrast to a previous study in non-growing patients, which showed that 1Z superimposition is a viable option when scans with smaller FOV are available [16]. The detected accuracy levels were similar to our AC and BZ superimpositions. However, the zygomatic arches were not influenced by treatment and/or growth, whereas BZ superimposition was not tested at all. On the contrary, the zygomatic arches were extensively altered by treatment in our patient sample [8,52,53]. The limited surface of one zygomatic arch might also influence results. From our data it cannot be concluded if inadequate superimposition was due to this reason, to the considerable change induced in this structure by treatment, or to a combination of the above. The duplicated pre-treatment models were perfectly registered also using one zygomatic arch. Furthermore, in the study of Nada et al. [16], the Bland-Altman or a similar method was not applied to the data to account for the limitations of the used tests (correlations or comparisons of means) for comparing methods of measurement [41,42]. In any case, until the clarification of this issue, we suggest the use of both zygomatic arches, since they are usually available in small FOV images.

### Efficacy of the superimposition technique

Similar techniques for comparisons of polygonal surface models have already been used for medical, dental, and orthodontic applications, such as for facial imaging [27,54] or to assist craniomaxillofacial reconstruction [55,56], but the validity of superimposition of serial 3D craniofacial scans is thoroughly tested for first time here.

The time required by all operators to complete the superimposition procedure and analysis was approximately 25 minutes. This is shorter than in previous reports [15,16] and is mostly required for computational reasons (15 min). Thus, it will be much shorter in the near future due to the rapid progress of hardware systems.

Another advantage of this technique is that the operator does not have to select exactly the same reference area in the two models. Only a principal anatomical area is required. Even the extent of the selected areas can differ. With the iterative closest point approach point pairs are rejected if one of the points lies at the boundary of the mesh (or the selected region). There is actually no need to select areas on both meshes; one can even select the area on one mesh. That area will ‘slide’ on the other mesh until convergence [28,29]. Thus, the accuracy of the technique is not depending on the operator and on proper landmark/area identification [47]. For the needs of our study, the operators selected areas on both meshes to decrease running time. They were also instructed to select similar reference areas between different models and patients for consistency reasons.

A promising alternative technique that could overcome the need for a single reference structure might be a landmark-based geometric morphometric approach [57,58]. With this approach, one can superimpose sets of landmarks that describe the shape of objects, instead of the whole objects. A major advantage is that the results of the statistical analyses of landmark coordinates can be visualised as shapes or shape deformations [57]. Furthermore, superimposition of quite different objects is possible with this approach, though this is not usually the case in clinical practice. In surface-based or voxel-based approaches, superimposition of quite different objects may be confounded by the automated transformation models used for image registration. That is because in such cases the registered points/voxels may not necessarily be homologous or corresponding after the superimposition [5]. However, implementation of geometric morphometric approaches requires proper identification of a number of landmarks. Though extremely useful for certain research questions [57,59], the significant operator time required, along with the need for accurate and reproducible landmark identification [18,19], make their use less convenient than surface- or voxel-based approaches. In any case, the scientific question should define the type of registration that best fits the data.

The accuracy of each superimposition technique was determined by the congruence between specific corresponding structures. Accuracy could have been assessed through the calculation of the total deviation (D) of corresponding structures or the deviation in any of the three planes of space (x, y, z). We used the accuracy of D, as a value indicative of accuracy in all three planes, since high inaccuracy in any of the three levels would not be acceptable anyway. The mean accuracy of superimposition techniques for 3D datasets varies among studies [6,8,16,52,60] between 0.13 and 1.5 mm. By setting the minimum acceptable distance between corresponding structures at 0.3 mm, we attempted to define the acceptable level of accuracy, but this was not always feasible. For our gold standard technique (AC + F), the accuracy of superimposition ranged between 0.04 and 0.17 mm, which is quite satisfying particularly when considering that the CT slice thickness was 0.8 mm. The AC and BZ superimpositions also provided acceptable values that were below 0.5 mm in most cases, even though the zygomatic structures were changed considerably by the orthodontic intervention itself (between T0 and T1). Continuing improvement of radiological imaging techniques will undoubtedly lead to improvement of resolution and subsequent increase in superimposition accuracy and precision without exposing the patient to large amounts of radiation [47].

To our knowledge, by using retrospectively obtained CT data, this study was the first to compare superimposition on a number of reference structures with the present methodology. We provided guidelines for optimal superimposition of serial 3D datasets, applicable even to patients with extended skeletal changes induced by treatment, and when using a reduced FOV. Exposure to radiation would be even more reduced if CBCT scans were used instead of CT [61]. This is definitely what we suggest as the standard clinical practice when 3D information is necessary for diagnosis and/or treatment planning and not as a routine procedure [24]. The applicability and the order of validity of the proposed superimposition techniques is not expected to change considerably when applied to CBCT data [47]. However, this remains to be tested, since segmentation of bone in a CBCT is not as simple as in a CT (CBCTs do not have consistent voxel densities and HUs do not apply) and this may consist an additional source of error.

## Conclusions

Superimposition of 3D surface models created from voxel data can provide accurate, precise, and reproducible results even in patients with extensive changes of craniofacial structures induced by treatment. Using the technique described here, quantification of structural changes on the superimposed models is easily feasible. Communication between scientists is also improved due to the increased post-processing capabilities. In our study population, the superimposition on both zygomatic arches was comparable to the superimposition on the anterior cranial base, although it presented slightly higher levels of random error. Its main advantage is the applicability to scans with a smaller field of view, which require much less radiation dose.

## Supporting Information

S1 Table Non parametric MANOVA on accuracy measurements (deviation between structures). Three crossed factors and their possible interactions were analyzed: superimposition technique (fixed factor; 5 techniques), operator (random factor; 3 operators), and time (fixed factor; 2 time points). Data were transformed to fourth-root. Analysis was based on Bray-Curtis distances. Unrestricted permutation of raw data using correct permutable units was performed. No. of permutations used = 9999. R2 = 0.61%.
(DOCX)

S2 Table Non parametric MANOVA on accuracy measurements (deviation between structures). Three crossed factors and their possible interactions were analyzed: superimposition technique (fixed factor; 5 techniques), operator (random factor; 3 operators), and time (fixed factor; 2 time points). Data were transformed to fourth-root. Analysis was based on Bray-Curtis distances. Permutation of residuals under the reduced model was performed. No. of permutations used = 9999. R2 = 0.61%.
(DOCX)

S3 Table Inter-operator reliability of each superimposition technique. This was calculated for the distances (x, y, z, D) between the two serial datasets (T0, T1) at 4 specific points, measured by each operator in each patient (n = 8 patients x 4 points x 4 values for each point = 128; significance level 0.01; Pearson’s correlation). From this analysis, it can be concluded that inter-operator reliability was high for the 3-point registration technique and excellent for the other four techniques.
(DOCX)

S4 Table Overall distance (D) of two serial datasets at four specific points. These distances were measured by each operator following each superimposition technique (n = 8 patients x 1 point x 1 value = 8; significance level 0.01). The generalized linear model with repeated measures was used for comparisons. From this analysis, it can be concluded that the precision of all registration techniques was high since no significant differences were detected between operators on directly measured structural changes on the superimposed models, at four pre-defined points, concerning each one of the superimposition techniques.
(DOCX)
