# Supplementary material for: Evaluation of 3-Dimensional Superimposition Techniques on Various Skeletal Structures of the Head Using Surface Models
Source: PLoS One. 2015 Feb 23;10(2):e0118810. doi: 10.1371/journal.pone.0118810 (PMC4338241; doi:10.1371/journal.pone.0118810)
Supplement: S1 Table — Three crossed factors and their possible interactions were analyzed: superimposition technique (fixed factor; 5 techniques), operator (random factor; 3 operators), and time (fixed factor; 2 time points). Data were transformed to fourth-root. Analysis was based on Bray-Curtis distances. Unrestricted permutation of raw data using correct permutable units was performed. No. of permutations used = 9999. R2 = 0.61%. (DOCX) [file pone.0118810.s001.docx]

**Table S1.** **Non parametric MANOVA on accuracy measurements (deviation between structures).**

| **Source** | **d.f.** | **SS** | **MS** | | **F** | **p** |
| --- | --- | --- | --- | --- | --- | --- |
| Superimposition | 4 | 24822.88 | 6205.72 | | 164.64 | 0.0002* |
| Operator | 2 | 132.46 | 66.23 | | 0.80 | 0.4508 |
| Time | 1 | 444.54 | 444.54 | | 34.65 | 0.0108 |
| Superimposition x Operator | 8 | 301.53 | 37.69 | | 0.46 | 0.9014 |
| Superimposition x Time | 4 | 774.32 | 193.58 | | 5.09 | 0.0221* |
| Operator x Time | 2 | 25.66 | 12.83 | | 0.16 | 0.8922 |
| Superimposition x Operator x Time | 8 | 304.19 | 38.02 | | 0.46 | 0.9037 |
| Residual | 210 | 17324.18 | 82.50 | |  |  |
| Total | 239 | 44129.76 |  | |  |  |
| **Comparison**^1^ | **t** | | | **p** | | |
| 3P vs. AC | 9.45 | | | 0.0006* | | |
| 3P vs. AC + F | 22.82 | | | 0.0001* | | |
| 3P vs. BZ | 5.61 | | | 0.0053 | | |
| 3P vs. 1Z | 6.37 | | | 0.0039* | | |
| AC vs. AC + F | 14.48 | | | 0.0001* | | |
| AC vs. BZ | 0.89 | | | 0.4250 | | |
| AC vs. 1Z | 14.32 | | | 0.0003* | | |
| AC + F vs. BZ | 8.67 | | | 0.0009* | | |
| AC + F vs. 1Z | 26.33 | | | 0.0001* | | |
| BZ vs. 1Z | 7.83 | | | 0.0007* | | |

Three crossed factors and their possible interactions were analyzed: superimposition technique (fixed factor; 5 techniques), operator (random factor; 3 operators), and time (fixed factor; 2 time points). Data were transformed to fourth-root. Analysis was based on Bray-Curtis distances. Unrestricted permutation of raw data using correct permutable units was performed. No. of permutations used = 9999. R^2^ = 0.61%.

^1^Pair-wise *a posteriori* tests among superimposition techniques using the *t*-statistic (p < 0.005; Bonferroni correction applied; Monte Carlo asymptotic p-value). Operators and time points were ignored in the pair-wise tests.

* denotes statistical significance.

3P: three-point registration; AC: anterior cranial base; AC + F: anterior cranial base + foramen magnum; BZ: both zygomatic arches; 1Z: one zygomatic arch
