# Supplementary material for: Evaluation of 3-Dimensional Superimposition Techniques on Various Skeletal Structures of the Head Using Surface Models
Source: PLoS One. 2015 Feb 23;10(2):e0118810. doi: 10.1371/journal.pone.0118810 (PMC4338241; doi:10.1371/journal.pone.0118810)
Supplement: S3 Table — This was calculated for the distances (x, y, z, D) between the two serial datasets (T0, T1) at 4 specific points, measured by each operator in each patient (n = 8 patients x 4 points x 4 values for each point = 128; significance level 0.01; Pearson’s correlation). From this analysis, it can be concluded that inter-operator reliability was high for the 3-point registration technique and excellent for the other four techniques. (DOCX) [file pone.0118810.s003.docx]

**Table S3. Inter-operator reliability of each superimposition technique**.

|  | **Operators** | | |
| --- | --- | --- | --- |
|  | **1 and 2**  **(r)** | **2 and 3**  **(r)** | **1 and 3**  **(r)** |
| **3P** | 0.77*** | 0.76*** | 0.79*** |
| **AC** | 0.95*** | 0.98*** | 0.94*** |
| **AC + F** | 0.98*** | 0.99*** | 0.98*** |
| **BZ** | 0.96*** | 0.91*** | 0.92*** |
| **1Z** | 0.99*** | 0.98*** | 0.98*** |

This was calculated for the distances (x, y, z, D) between the two serial datasets (T0, T1) at 4 specific points, measured by each operator in each patient (n = 8 patients x 4 points x 4 values for each point = 128; significance level 0.01; Pearson’s correlation). From this analysis, it can be concluded that inter-operator reliability was high for the 3-point registration technique and excellent for the other four techniques.

3P: three-point registration; AC: anterior cranial base; AC + F: anterior cranial base + foramen magnum; BZ: both zygomatic arches; 1Z: one zygomatic arch

***p<0.001
