# Supplementary material for: Evaluation of 3-Dimensional Superimposition Techniques on Various Skeletal Structures of the Head Using Surface Models
Source: PLoS One. 2015 Feb 23;10(2):e0118810. doi: 10.1371/journal.pone.0118810 (PMC4338241; doi:10.1371/journal.pone.0118810)
Supplement: S4 Table — These distances were measured by each operator following each superimposition technique (n = 8 patients x 1 point x 1 value = 8; significance level 0.01). The generalized linear model with repeated measures was used for comparisons. From this analysis, it can be concluded that the precision of all registration techniques was high since no significant differences were detected between operators on directly measured structural changes on the superimposed models, at four pre-defined points, concerning each one of the superimposition techniques. (DOCX) [file pone.0118810.s004.docx]

**Table S4. Overall distance (D) of two serial datasets at four specific points.**

| **Point 1 (mean±SD)** | | | | | |
| --- | --- | --- | --- | --- | --- |
|  | **3P** | **AC** | **AC + F** | **BZ** | **1Z** |
| **Operator 1** | 2.68+1.09 | 2.23+0.94 | 2.02+0.67 | 1.60+0.72 | 3.85+1.20 |
| **Operator 2** | 2.23+1.17 | 2.00+0.43 | 1.99+0.57 | 1.80+0.90 | 3.93+1.27 |
| **Operator 3** | 2.47+1.32 | 2.04+0.74 | 1.83+0.72 | 1.98+0.91 | 3.82+1.31 |
| **p-value** | 0.633 | 0.565 | 0.204 | 0.205 | 0.713 |
| **Point 2 (mean±SD)** | | | | | |
|  | **3P** | **AC** | **AC + F** | **BZ** | **1Z** |
| **Operator 1** | 1.56+0.88 | 2.26+0.78 | 2.11+0.66 | 2.00+0.86 | 1.02+0.42 |
| **Operator 2** | 2.09+1.07 | 1.99+0.80 | 1.88+0.77 | 2.06+1.14 | 1.29+0.43 |
| **Operator 3** | 2.62+1.99 | 2.12+0.73 | 1.98+0.73 | 2.31+1.20 | 1.31+0.72 |
| **p-value** | 0.185 | 0.314 | 0.164 | 0.377 | 0.403 |
| **Point 3 (mean±SD)** | | | | | |
|  | **3P** | **AC** | **AC + F** | **BZ** | **1Z** |
| **Operator 1** | 3.59+1.43 | 3.47+0.91 | 3.18+0.54 | 2.54+0.53 | 5.38+1.76 |
| **Operator 2** | 3.54+1.65 | 3.32+0.95 | 3.21+0.54 | 2.56+0.71 | 5.62+1.71 |
| **Operator 3** | 3.66+1.25 | 3.40+1.07 | 3.13+0.53 | 3.22+1.05 | 5.45+1.88 |
| **p-value** | 0.972 | 0.842 | 0.564 | 0.142 | 0.229 |
| **Point 4 (mean±SD)** | | | | | |
|  | **3P** | **AC** | **AC + F** | **BZ** | **1Z** |
| **Operator 1** | 3.60+2.32 | 3.84+1.02 | 3.51+0.88 | 3.34+1.27 | 2.06+0.89 |
| **Operator 2** | 3.38+1.73 | 3.77+1.25 | 3.56+0.93 | 3.46+1.30 | 2.10+0.93 |
| **Operator 3** | 3.84+1.68 | 3.72+1.33 | 3.52+0.96 | 3.72+1.17 | 2.00+0.98 |
| **p-value** | 0.636 | 0.903 | 0.710 | 0.424 | 0.632 |

These distances were measured by each operator following each superimposition technique (n = 8 patients x 1 point x 1 value = 8; significance level 0.01). The generalized linear model with repeated measures was used for comparisons. From this analysis, it can be concluded that the precision of all registration techniques was high since no significant differences were detected between operators on directly measured structural changes on the superimposed models, at four pre-defined points, concerning each one of the superimposition techniques.

3P: three point registration; AC: anterior cranial base; AC + F: anterior cranial base + foramen magnum; BZ: both zygomatic arches; 1Z: one zygomatic arch
